# 非小细胞肺癌*EGFR*外显子20插入突变检测和靶向药物研究进展

**DOI:** 10.3779/j.issn.1009-3419.2023.102.06

**Published:** 2023-02-20

**Authors:** Yulu WANG, Tianqing CHU

**Affiliations:** ^1^200030 上海，上海交通大学附属胸科医院病理科（王钰璐）; ^1^Department of Pathology; ^2^呼吸内科（储天晴）; ^2^Department of Respiratory Medicine, Shanghai Chest Hospital, Shanghai Jiao Tong University, Shanghai 200030, China

**Keywords:** 表皮生长因子受体外显子20插入突变, 检测方法, 靶向治疗, 肺肿瘤, Epidermal growth factor receptor ex20ins mutation, Detection method, Target therapy, Lung neoplasms

## Abstract

表皮生长因子受体外显子20插入（epidermal growth factor receptor exon 20 insertion, EGFR ex20ins）突变是最早被发现的非小细胞肺癌（non-small cell lung cancer, NSCLC）驱动基因激活突变之一。由于这种突变引起的蛋白变异的独特结构，大多数EGFR ex20ins突变（除A763_Y764insFQEA）患者对已上市的第一、二、三代EGFR酪氨酸激酶抑制剂（EGFR-tyrosine kinase inhibitors, EGFR-TKIs）治疗应答较差。随着针对EGFR ex20ins的新型靶向药物经美国食品药品监督管理局（Food and Drug Administration, FDA）及其他国家注册部门相继获批，国内针对EGFR ex20ins的靶向药物研发迅速发展，针对EGFR ex20ins的靶向药物莫博赛替尼也于近日在国内正式获批。EGFR ex20ins是一类分子异质性很强的变异类型，如何在临床实践中全面精准地检出，让越来越多的患者从靶向治疗中获益，是一个值得关注、亟需解答的问题。本文对EGFR ex20ins的分子分型、检测的重要性及不同检测方法的差异性进行介绍，并归纳了以EGFR ex20ins为靶向的新药研发进展，以期通过选择精准、快速、合适的检测方法，优化EGFR ex20ins患者的诊疗路径，从而改善患者的临床获益。

## Abstract (en)

Epidermal growth factor receptor exon 20 insertion (EGFR ex20ins) is one of the earliest driver gene activation mutations in non-small cell lung cancer (NSCLC). However, due to the unique structure of protein variation caused by this mutation, most patients with EGFR ex20ins mutation (except A763_Y764insFQEA) have poor response to the launched first/second/third generation EGFR-tyrosine kinase inhibitors (EGFR-TKIs). With the successive approval of new specific targeted drugs for EGFR ex20ins in Food and Drug Administration (FDA) and other national regulatory agencies, the development and clinical research of targeted drugs for EGFR ex20ins in China have also developed rapidly and Mobocertinib has been approved recently in China. It is worth noting that EGFR ex20ins is a variant type with strong molecular heterogeneity. How to detect it comprehensively and accurately in clinical practice, so as to enable more patients to benefit from targeted therapy, is a very important and urgent problem to be solved. This review introduces the molecular typing of EGFR ex20ins, then discusses the importance of EGFR ex20ins detection and the differences of various detection methods, and summarizes the research and development of new drugs progress of EGFR ex20ins, in order to optimize the diagnosis and treatment path of EGFR ex20ins patients by selecting accurate, rapid and appropriate detection methods, so as to improve the clinical benefits of the patients.

2020年世界卫生组织国际癌症研究机构（International Agency for Research on Cancer, IARC）发布了全球新发癌症数据，中国新发癌症457万例，占全球23.7%，其中肺癌新增人数为220万例，死亡180万例，位居癌症死亡人数第一，远超其他肿瘤类型。从小细胞肺癌、非小细胞肺癌（non-small cell lung cancer, NSCLC）内科化疗^[1]^，到基于组织类型的NSCLC不同亚型治疗^[2⇓-4]^，再到靶向治疗^[5]^、免疫治疗^[6,7]^，我国肺癌患者的中位生存期已从传统化疗时代的10个多月，到目前延长近3年，其中针对某些肺癌亚型的靶向治疗甚至超过5年，疗效有了很大提升^[8]^。目前肺癌常规检测靶点有表皮生长因子受体（epidermal growth factor receptor, EGFR）、间变性淋巴瘤激酶（anaplastic lymphoma kinase, ALK）、c-ros肉瘤致癌因子-受体酪氨酸激酶（ROS proto-oncogene 1, receptor tyrosine kinase, ROS1）、Kirsten大鼠肉瘤病毒癌基因同源物（Kirsten rat sarcoma viral oncogene homolog, KRAS）、转染重排（rearranged during transfection, RET）、V-raf鼠肉瘤病毒癌基因同源物B1（V-raf murine sarcoma viral oncegene homolog B1, BRAF）、间质-上皮细胞转化因子（mesenchymal-epithelial transition factor, MET）、神经营养因子受体络氨酸激酶（neurotrophin receptor kinase, NTRK）、人表皮生长因子受体2（human epidermal growth factor receptor 2, HER2）、程序性死亡配体1（programmed cell death ligand 1, PD-L1）等基因，针对这些靶点的多种治疗药物均已进入临床应用或正在开展临床试验^[9]^。

EGFR基因位于人7号染色体p12-q22区，包括28个外显子。EGFR激活突变通常出现在EGFR酪氨酸激酶结构域的前4个外显子（外显子18、19、20和21）。EGFR突变在40%-50%的亚洲NSCLC患者和10%-30%的白种人患者中均可检测到^[10,11]^，中国NSCLC患者中的EGFR基因突变和扩增的发生率明显高于西方人群^[12]^。EGFR突变除常见的L858R、EGFR外显子19缺失（EGFR exon 19 deletion, EGFR ex19del）、EGFR外显子20插入（EGFR exon 20 insertion, EGFR ex20ins）和T790M之外，还有很多不同种类的罕见突变^[12]^。EGFR突变NSCLC患者中EGFR ex20ins的发生率为4%-12%，而在NSCLC患者中，发生率为1.8%-2.3%^[13,14]^。EGFR ex20ins主要通过框内复制和/或插入导致的突变，是仅次于EGFR ex19del和EGFR L858R的第三种最为常见的EGFR突变类型。EGFR ex20ins突变在腺癌、女性、从不吸烟和亚洲患者中更常见^[15]^。

事实上，作为发现最早的NSCLC驱动基因激活突变之一^[16,17]^，EGFR ex20ins突变会引起蛋白变异的独特结构，导致大多数EGFR ex20ins突变（除A763_Y764insFQEA）患者对第一、二、三代EGFR酪氨酸激酶抑制剂（EGFR-tyrosine kinase inhibitors, EGFR-TKIs）治疗均应答较差^[18]^。随着EGFR ex20ins的新型靶向药物，包括JNJ-61186372（Amivantamab）和Mobocertinib（莫博赛替尼），在美国食品药品监督管理局（Food and Drug Administration, FDA）及其他国家注册部门相继获批^[19⇓-21]^，国内针对EGFR ex20ins突变为靶向的药物开发和临床研究也快速发展起来^[22⇓⇓-25]^。值得注意的是，EGFR ex20ins具有很强的分子异质性，目前已有超过100种EGFR ex20ins变异类型的报道^[22,25]^。因此，如何选择更合适的检测方法，以全面准确地检测出EGFR ex20ins突变，是临床实践中亟待解决的问题。

本文对EGFR ex20ins的分子分型、检测的重要性及不同检测方法的差异性进行介绍，并归纳了以EGFR ex20ins为靶向的新药研发进展，以期优化EGFR ex20ins患者的诊疗路径，从而改善患者临床获益。

## 1 EGFR ex20ins的分子分型

EGFR外显子20负责转录E762-K823位置的氨基酸，包括由D761-M766氨基酸构成的C-螺旋以及由A767-C775氨基酸构成的环^[26]^，大部分EGFR ex20ins突变均位于C-螺旋及其之后的环内，最常见的突变位点是D770-N771的插入，其次是V769-D770的插入（图1）。在D761-M766的插入称为前端插入，A767-C775的插入称为后端插入。在后端插入中又提出了近环插入（A767-P772）和远环插入（H773-C775）的概念^[27]^。一项国内的回顾性研究结果（n=24,468）^[28]^表明中国NSCLC患者中EGFR ex20ins的突变率为2.24%，共发现85种突变形式，主要有A767_V769dup、S768_D770dup、A763_Y764insFQEA、N771_H773dup、H773_V774dup、D770_N771insG、D770delinsGY、H773dup、P772_H773dup和H773_V774insAH等突变类型，而Xu等^[29]^报道的晚期NSCLC患者携带EGFR ex20ins的突变率为1.3%，有32种亚型，其中A767_V769dupASV、S768_D770dupSVD、N771_H773dupNPH、H773_V774dupHV、H773delinsPNPY、D770delinsGY和P772_H773dupPH等为主要突变类型。表1的数据说明携带EGFR ex20ins突变的NSCLC患者中，突变亚型出现频率最高的是A767_V769dupASV（V769_D770insASV）、N771_H773dupNPH（H773_V774insNPH）和S768_D770dupSVD（D770_N771insSVD）^[28⇓-30]^。

**图 1:**
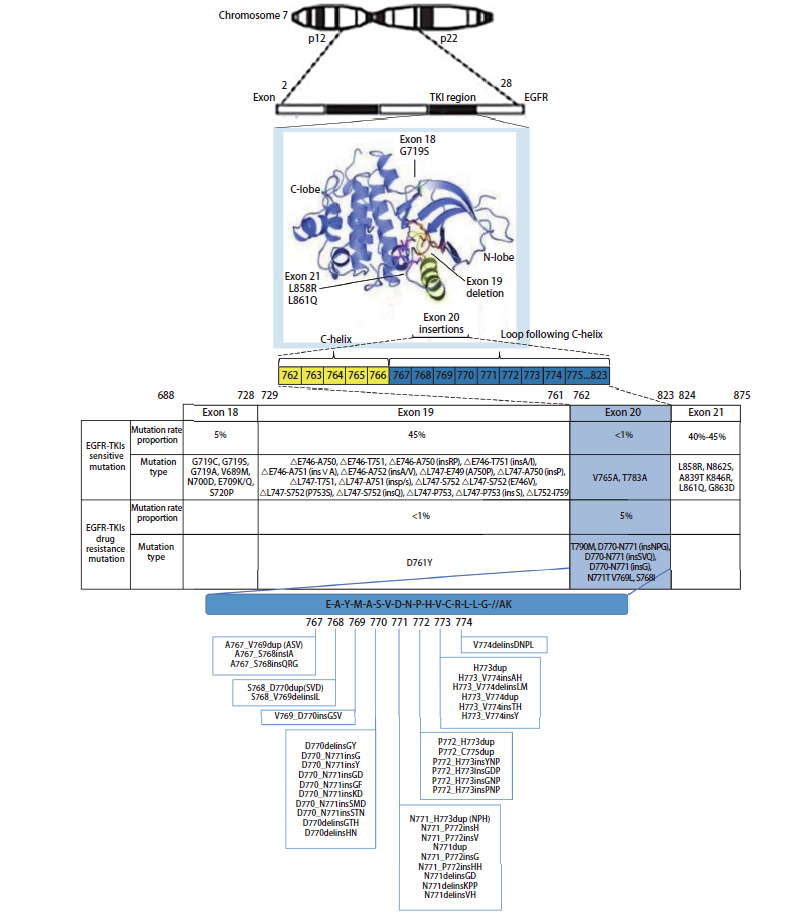
EGFR ex20ins的分子结构和分子亚型（源自于蛋白质结构数据库PDB ID 1XKK）

**表1:** 在NSCLC中EGFR ex20ins的检测方法、发生率、突变亚型及突变频率文献数据整理

Detection methods	EGFR ex20ins incidence rate	Subtypes	Mutation frequency	Ref.
NGS	2.24% (547/24,468)	85 subtypes		^[28]^
		A767_V769dup	26.51%	
		S768_D770dup	17.55%	
		A763_Y764insFQEA，N771_H773dup，H773_V774dup	The mutation frequency of each subtype was 4.75%	
		D770_N771insG	4.39%	
		D770delinsGY	3.84%	
		H773dup	3.29%	
		P772_H773dup	2.93%	
		H773_V774insAH	2.19%	
		Others	25.05%	
NGS	Advanced NSCLC patients (IIIB-IV) 1.30% (119/9,142)	32 subtypes		^[28,29]^
		A767_V769dupASV	27.73%	
		S768_D770dupSVD	15.97%	
		N771_H773dupNPH	9.24%	
		H773_V774dupHV	5.04%	
		H773delinsPNPY, D770delinsGY	The mutation frequency of each subtype was 4.20%	
		P772_H773dupPH	3.36%	
		H773_V774insTH, D770_N771insG，S768_V769insIL	The mutation frequency of each subtype was 2.52%	
		A763_Y764insFQEA, D770_P772dupDNP, N771delinsKG, N771_P772insH, H773_V774insAH	The mutation frequency of each subtype was 1.68%	
		M766_A767insASV, A767_S768insASVD, S768_V769insLDS, V769_D770insDNP, D770delinsGT, D770delinsNNPH, D770_N771insH, D770_N771insNPY, N771dupN, N771delinsGT, N771delinsSH, N771_P772insT, P772_H773insGT, P772_H773insNPNP, P772_C775dupHH, H773delinsYPNPY, H773_V774insP	The mutation frequency of each subtype was 0.84%	
Sanger sequencing	0.38% (67/17,664)			^[30]^
		N771_H773dup, A767_V769dup	The mutation frequency of each subtype was 11.94%	
		S768_V769insIL, S768_D770dupSVD	The mutation frequency of each subtype was 8.96%	
		H773_V774dupH, D770_N774insSVD	The mutation frequency of each subtype was 5.97%	
		D770_N771insG	2.99%	
		E762_A763insVAS, V769_D770insAVS, V769_D770insSSV, V769_D770insLDNPH, D770_XXXdelinsGI, N771_P772insGF, N771_P772insGY, N771_P772insPHVR, P772_H773insQ, Tyr772_A775dup, P772_H773insHTW, P772_H773insHV, H773_V774insAH, H773_V774insAV, H773_V774delinsLMH773_V774delinsW, G778_P780dup	The mutation frequency of each subtype was 1.49%	
		Others	17.91%	
Detection methods	EGFR ex20ins incidence rate	Subtypes	Mutation frequency	Ref.
Sanger sequencing	2.49% (27/1,086)	13 subtypes		^[15]^
		V769_D770insASV	22.22%	
		H773insH, A763_Y764insFQEA	The mutation frequency of each subtype was 11.11%	
		H773_V774insPH, H773_V774insNPH, V774_C775insHV, D770_N771insSVD, D770delinsGY	The mutation frequency of each subtype was 7.41%	
		N771_P772insV, P772_H773insPNP, D770_N771insGL, N771delinsGY, H773_V774insAH	The mutation frequency of each subtype was 3.70%	

NGS: next generation sequencing; NSCLC: non-small cell lung cancer; PCR: polymerase chain reaction.

由于EGFR ex20ins突变亚型的氨基酸构型形成了一种异常结构，导致药物结合口袋体积显著减少，限制了和药物结合的区域，明显地降低了与针对经典突变的传统EGFR-TKIs结合的亲和力。因此，除了A763_Y764ins FQEA与传统EGFR-TKIs治疗的敏感性相关，A763_Y764ins LQEA与第一、三代EGFR-TKIs可能有敏感相关性，其他EGFR ex20ins突变位点对于针对经典突变的传统EGFR-TKIs应答普遍较差^[31]^。此外，作为EGFR ex20ins的靶向药物，波齐替尼的敏感性高度依赖于插入位置。研究^[27]^结果表明，在插入EGFR ex20ins后端的突变区域中，近环插入（A767-P772）较远环插入（H773-C775）敏感性更高[客观缓解率（objective response rate, ORR）分别为46%和0%，P=0.0015]。而其他以EGFR ex20ins为靶向的药物（如Amivantamab^[32]^、DZD9008^[33]^、CLN-081^[34]^）尚无一致结论明确插入位置与疗效的相关性。

## 2 EGFR ex20ins检测的重要性及检测方法差异分析

### 2.1 EGFR ex20ins突变检测的重要性

国内外指南或共识均强调EGFR ex20ins检测具有重要意义，推荐常规EGFR基因检测中应包括EGFR ex20ins突变（表2）^[5,30,34]^。聚合酶链反应（polymerase chain reaction, PCR）及下一代测序技术（next generation sequencing, NGS）是常用的检测方法，PCR可检测到的EGFR ex20ins突变类型有限，而NGS检测范围更加广泛全面^[31]^。考虑到EGFR ex20ins突变的高度异质性，欧洲肿瘤内科学会（European Society for Medical Oncology, ESMO）共识建议优先选择NGS检测EGFR ex20ins突变^[35]^。美国国立综合癌症网络（National Comprehensive Cancer Network, NCCN）指南同样明确推荐NGS为优先检测方法。中国临床肿瘤学会（Chinese Society of Clinical Oncology, CSCO）指南推荐EGFR突变检测方法包括突变扩增阻滞系统（amplification refractory mutation system, ARMS）PCR、Super ARMS PCR、Cobas® PCR、微滴式数字PCR（droplet digital PCR, ddPCR）和NGS等，但针对检测方法的选择尚无明确优先级推荐^[5]^。值得注意的是，CSCO指南并未对EGFR ex20ins和常见的EGFR敏感突变的检测方法进行区分，随着以EGFR ex20ins为靶标的药物获批，相信不久的将来，CSCO指南也会增加针对EGFR ex20ins检测方法的优先推荐建议。

**表2:** 国内外指南或共识中关于EGFR ex20ins突变检测推荐的关键点汇总

	Guideline	Key recommendation about the detection of EGFR ex20ins
Foreign	NCCN^[31]^	Because some EGFR ex20ins are or may be sensitive to first- and third-generation inhibitors, the insertion mutations remain important. Some assays will identify the presence of EGFR ex20ins, additional testing to further clarify the specific subtypes may be indicated for therapy. Targeted PCR-based approaches for detection of EGFR variants may under-detect EGFR ex20ins, NGS are preferred.
	ESMO^[35]^	With the emergence of specific targeted therapies, EGFR ex20ins mutations are therapeutically relevant. Given the high mutation heterogeneity and the difference in response about different mutations, NGS should be considered as a priority.
	CAP/IASLC/AMP^[41,42]^	Routine EGFR mutation testing should cover S768, T790M, and 20ins mutations in exons 20.
China	CSCO guidelines for diagnosis and treatment of NSCLC (2020)^[5]^	EGFR mutation testing should be performed routinely in all NSCLC with adenocarcinoma, regardless of clinical characteristics (such as smoking history, gender, ethnicity, or others). EGFR mutation testing should cover EGFR exons 18, 19, 20 and 21. Especially in the case of limited sample size, validated detection methods can be used to detect multiple driver genes at the same time, such as PCR or NGS for simultaneous detection of multiple genes.
	Clinical diagnosis and treatment guidelines for lung cancer of Chinese Medical Association (2019)^[43]^	EGFR mutation testing should cover EGFR exons 18, 19, 20, 21 (Class 1 recommendation evidence). Some types of EGFR mutations did not respond to first-generation EGFR-TKIs therapy (eg. ex20ins and p.T790M). However, EGFR ex20ins mutation (p.A763_Y764insFQEA) is still effective for EGFR-TKIs treatment, and specific mutation sites of ex20ins mutation need to be identified (Class 2A recommendation evidence).
	Guidelines on clinical practice of molecular tests in NSCLC in China^[44]^	No matter what detection methods are used (Sanger sequencing, qRT-PCR, second-generation sequencing, etc.), the most important mutation sites of EGFR should be included, including exon 20 insertion mutations (20ins), etc.
	Chinese expert consensus on next generation sequencing diagnosis for NSCLC (2020 edition)^[45]^	For patients with advanced new or postoperative recurrence of NSCLC, NPA-approved tests are recommended for initial testing, including at least the common NSCLC driver genes: EGFR mutations (which should cover exons 18, 19, 20, 21), ALK fusion and ROS1 fusion (Grade I recommendation).

NCCN: National Comprehensive Cancer Network; ESMO: European Society for Medical Oncology; CSCO: Chinese Society of Clinical Oncology; NPA: Natural Products Association; ALK: anaplastic lymphoma kinase; ROS1: recombinant C-Ros oncogene 1, receptor tyrosine kinase; qRT-PCR: quantitative real time polymerase chain reaction.

### 2.2 EGFR ex20ins不同检测方法差异分析

目前已获批的EGFR检测方法在国际上有实时PCR、NGS，可使用血浆或组织样本，但各检测方法可检测的基因数不同，且伴随诊断的药物也不同。针对EGFR ex20ins检测，FDA已批准两种伴随诊断。一个是基于组织样本的NGS检测平台Oncomine Dx Target Test，用于莫博赛替尼的伴随诊断^[36]^。一个是基于血浆样本的NGS检测平台Guardant 360 CDx，可用于Amivantamab的伴随诊断^[37]^。

国内目前暂无针对EGFR ex20ins的伴随诊断获批，但已有多款EGFR检测试剂盒获批，涉及PCR和NGS平台。基于PCR平台目前仅能覆盖最多15种EGFR ex20ins突变类型（表3），主要可检测A767_V769dupASV（V769_D770insASV）、N771_H773dupNPH（H773_V774insNPH）和S768_D770dupSVD（D770_N771insSVD）等亚型。但EGFR ex20ins突变类型已发现约100多种^[22,25]^，因此现阶段PCR尚无法满足EGFR ex20ins的覆盖需求。而已获批的NGS试剂盒相比PCR检测更为全面，可广泛覆盖EGFR ex20ins突变类型（表3）。此外，已有国外临床研究^[38]^和真实世界研究^[39]^发现PCR存在40%-50%的漏检率，而NGS可更好地提高EGFR ex20ins突变检出率。国内学者于JAMA Oncology发表的临床研究中已采用NGS为EGFR ex20ins检测方案^[40]^。由于国内外临床上常规使用的PCR平台存在较大的差异以及国内外人群EGFR ex20ins突变特征可能有所不同，真实世界研究中的PCR漏检情况有待国内学者的进一步探索。综上所述，一方面，亟需对现有PCR试剂盒进行升级迭代；另一方面，PCR检测EGFR ex20ins阴性的患者建议使用NGS进行复核，条件允许的情况下优先使用NGS检测EGFR ex20ins突变。

**表3:** 比较不同检测方法关于EGFR ex20ins的检测能力

Detection methods	Diagnosis company	Number of EGFR ex20ins subtypes covered by the detection methods	Specific subtypes
PCR	Cobas	5	V769_D770insASVor 767_769duplicate, D770_N771insSVD, D770_N771insG, H773_V774insH
	AmoyDx 9 in 1	15	H773_V774insH, D770_N771insG, V769_D770insASV, D770_N771insSVD, V769_D770insASV, H773_V774insNPH, H773_V774insQ, N771_P772insT, N771_P772insH, P772_H773insQ, H773_V774insV, V769_D770insGSV, D770_N771insG, D770_N771insG, P772_H773insDNP
	HEAS BioTech	3	V769_D770insASV, D770_N771insG, H773_V774insQ
	Tellgen	2	V769_D770insASV, D770_N771insG
NGS	BNS/BGI/AmoyDx/Nuohe/geneseeq/et al	All (Theoretically)	

## 3 EGFR ex20ins的药物治疗

在莫博赛替尼和Amivantamab获FDA批准用于EGFR ex20ins NSCLC之前，这部分患者主要的治疗方式有传统EGFR-TKIs、含铂化疗和免疫治疗^[5,31]^。但是，这些治疗方案获益有限。随着多种以EGFR ex20ins为靶点的药物问世，EGFR ex20ins NSCLC的生存和预后将得到显著改善。

### 3.1 传统的EGFR-TKIs、化疗和免疫治疗

EGFR ex20ins突变位置在α-C螺旋的旋转支点，插入的残基可能在空间上抑制C-螺旋的重定向，导致药物结合口袋体积显著减少并呈现失活特征。因此，EGFR ex20ins NSCLC患者对大多数传统EGFR-TKIs高度不应答或易耐药^[26]^。而化疗、免疫治疗也远不能满足EGFR ex20ins NSCLC患者的临床需求。一篇系统综述^[46]^汇总了全球19项临床试验和23项真实世界研究的数据，并根据治疗线程进行了荟萃分析。对于一线EGFR ex20ins患者，EGFR-TKIs的汇总ORR仅有6.8%，免疫治疗汇总ORR仅为14.0%。化疗汇总ORR最高，达到25.7%。化疗的汇总无进展生存期（progression-free survival, PFS）优于EGFR-TKIs或免疫治疗，分别为5.6个月、3.0个月和4.3个月。此外，化疗的汇总总生存期（overall survival, OS）最长，为18.3个月。对于二线患者，EGFR-TKIs、免疫治疗和化疗汇总ORR分别为5%、3.3%和13.9%，汇总PFS分别为2.1个月、2.3个月和4.4个月，汇总OS分别为14.1个月、8.8个月和17.1个月。

高剂量的第三代TKIs对于EGFR ex20ins NSCLC患者有一定的疗效潜力（表4）。ECOG-ACRIN 5162（NCT03191149）研究中，奥希替尼（160 mg, qd）用于EGFR ex20ins且既往经治的晚期NSCLC受试者。研究结果表明确认ORR（confirmed ORR, cORR）为25%（5/20），中位PFS为9.7个月（95%CI: 4.07-NA）^[47]^。POSITION20研究也探索了奥希替尼在一线EGFR ex20ins晚期NSCLC中的疗效。受试者接受奥希替尼（160 mg, qd）治疗。在25例受试者中ORR为28%（95%CI: 12%-49%），中位PFS为6.8个月（95%CI: 4.6-9.1），中位OS为15.2个月（95%CI: 14.3-16.0）^[48]^。此外，一项Ib期研究（FAVOUR 1, NCT04858958）探索了伏美替尼在EGFR ex20ins晚期NSCLC患者的临床疗效。2021年ESMO公布数据^[49]^显示，一线EGFR ex20ins晚期NSCLC受试者接受伏美替尼240 mg qd，ORR率为70%。因入组受试者人数较少，有待大样本研究进一步验证第三代EGFR-TKIs在EGFR ex20ins NSCLC患者的有效性和安全性。

**表4:** 正在研发或已获批EGFR外显子20插入突变靶向药物临床研究汇总

Type	Stage	Drug name	Trial registration number	Phase	Regimen	Enrolled population	Primary efficacy endpoint and outcome	Additional important efficacy outcome	Ref.
EGFR Ex20ins target	Approved indication study/Phase III clinical drug	TAK-788, Mobocertinib	NCT02716116	II	Mobocertinib 160 mg qd until progressive disease	Advanced NSCLC patients with EGFR ex20ins, who received platinum-based chemotherapy	cORR (IRC): 28%	cORR (INV): 35%; DCR (IRC): 78%; DOR (IRC): 15.8 mon; PFS (IRC): 7.3 mon; OS: 20.2 mon	^[51]^
			NCT04129502	III	Mobocertinib (160 mg qd) vs Platinum-based chemotherapy	Advanced NSCLC with EGFR ex20ins, first-line treatment	PFS (IRC) (Unpublished)	-	^[53]^
		Amivantamab	NCT02609776	I	Amivantamab 1,050 mg (baseline weight <80 kg) or 1,400 mg (baseline weight≥80 kg)	Advanced NSCLC patients with EGFR ex20ins, who received platinum-based chemotherapy	cORR (IRC): 39.5%	cORR (INV): 35.8% DOR (IRC): 11.1 mon; PFS (IRC): 8.3 mon; OS: 22.8 mon	^[55]^
			NCT04538664	III	Amivantamab+ platinum-based chemotherapy vs Platinum-based chemotherapy	Advanced NSCLC with EGFR ex20ins, first-line treatment	PFS (IRC) (Unpublished)	-	^[56]^
	Phase I/II clinical drug	Poziotinib	NCT03318939	II	Poziotinib 16 mg qd until progressive disease	Advanced NSCLC patients with EGFR ex20ins, who received platinum-based chemotherapy (cohort 1)	ORR: 14.8%	DCR: 68.7%; DOR: 7.4 mon; PFS: 4.2 mon	^[58]^
						Advanced NSCLC with EGFR ex20ins, first-line treatment (cohort 3)	cORR: 27.8%	DCR: 86.1%; DOR: 6.5 mon; PFS: 7.2 mon	^[59]^
		Sunvozertinib (DZD9008)	CTR20211009	II	Sunvozertinib 300 mg qd	Advanced NSCLC patients with EGFR ex20ins, who received platinum-based chemotherapy	ORR (BICR): 59.8%	-	^[60]^
		TAS6417 (CLN-081)	NCT04036682	I/II	TAS6417	Advanced NSCLC patients with EGFR ex20ins, who received platinum-based chemotherapy	cPR rate: 38.4%	DOR: 10 mon; PFS: 10 mon	^[62]^
		AP-L1898	NCT04993391	I/II	AP-L1898	Advanced NSCLC with EGFR ex20ins	ORR (Unpublished)	-	^[64]^
		HS-10376	NCT05435274	I/II	HS-10376	Previously treated, advanced NSCLC with EGFR ex20ins	ORR (Unpublished)	-	^[64]^
		BLU-451	NCT05241873	I/II	BLU-451	Advanced NSCLC with EGFR ex20ins	ORR (Unpublished)	-	^[64]^
		JS 111	NCT04993391	I/II	JS 111	Previously treated, advanced NSCLC with EGFR ex20ins	ORR (Unpublished)	-	^[64]^
		FWD1509	NCT05068024	I/II	FWD1509	Previously treated, advanced NSCLC with HER2/EGFR ex20ins	ORR (Unpublished)	-	^[64]^
		BAY 2927088	NCT05099172	I	BAY 2927088	Previously treated, advanced NSCLC with HER2/EGFR ex20ins	ORR (Unpublished)	-	^[64]^
		PLB1004	NCT05347628	I	PLB1004	Advanced NSCLC with HER2/EGFR ex20ins	ORR (Unpublished)	-	^[64]^
		YK-029A	CTR20180350	I	YK-029A	Advanced NSCLC with HER2/EGFR ex20ins	ORR (Unpublished)	-	^[37]^
Type	Stage	Drug name	Trial registration number	Phase	Regimen	Enrolled population	Primary efficacy endpoint and outcome	Additional important efficacy outcome	Ref
Traditional EGFR-TKIs	-	Osimertinib	NCT03191149	II	Osimertinib 160 mg qd	Previously treated, advanced NSCLC with EGFR ex20ins	cORR: 25%	PFS: 9.7 mon	^[47]^
			NL6705	II		Advanced NSCLC with EGFR ex20ins, first-line treatment	ORR: 28%	PFS: 6.8 mon OS: 15.2 mon	^[48]^
		Furmonertinib	NCT04858958	I	Furmonertinib 240 mg qd	Advanced NSCLC with EGFR ex20ins, first-line treatment	ORR: 70%	-	^[49]^
		Afatinib	NCT03727724	II	Afatinib 40 mg QD, cetuximab 500 mg/m² q2w	Advanced NSCLC with EGFR ex20ins	18-week DCR rate: 59%	ORR: 47% PFS: 5.5 mon	^[50]^
Other therapies	-	NVP-AUY922	NCT01854034	II	AUY922 70 mg/m^2^ qw	Previously treated, advanced NSCLC with EGFR ex20ins	ORR: 17%	-	^[63]^
		Tarloxotinib	NCT03805841	II	Tarloxotinib	Previously treated, advanced NSCLC with EGFR ex20ins	ORR (Unpublished)	-	^[64]^

ORR: objective response rate; IRC: Independent Review Committee; INV: Investigator; cORR: confirmed objective response rate; BICR: blinded independent central review; cPR: confirmed partial response; DCR: disease control rate; qd: once a day; q2w: once every two weeks; EGFR: epidermal growth factor receptor; TKIs: tyrosine kinase inhibitors; PFS: progression-free survival; OS: overall survival; DOR: duration of response.

西妥昔单抗联合EGFR-TKIs治疗EGFR ex20ins方案在NSCLC患者中进行了探索（表4）。AFACET研究（NCT03727724）中，EGFR ex20ins晚期NSCLC接受阿法替尼（40 mg, qd）联合西妥昔单抗（500 mg/m², q2w），结果表明在17例受试者中，18周的疾病控制率（disease control rate, DCR）为59%，ORR率为47%，中位PFS为5.5个月^[50]^。基于现阶段阿法替尼联合西妥昔单抗疗效数据的局限性，该方案的有效性有待进一步探索与验证。

### 3.2 EGFR ex20ins NSCLC的治疗新策略

近年来，针对EGFR ex20ins的特异性靶向药物的研发取得了飞速发展，多种EGFR ex20ins靶向药物在国内外展开了一系列临床研究并取得较好结果（表4）。

#### 3.2.1 获批适应证的研究/正在开展的III期临床研究

##### 3.2.1.1 莫博赛替尼

莫博赛替尼是口服、不可逆、高选择性的EGFR/HER2 ex20ins抑制剂。EXCLAIM（NCT02716116）是一项莫博赛替尼治疗EGFR ex20ins且既往接受含铂化疗NSCLC的I期/II期临床研究^[51]^（表4）。受试者接受莫博赛替尼（160 mg, qd）治疗，直到疾病出现进展或毒性无法耐受等。主要疗效终点为独立评审委员会（Independent Review Committee, IRC）评估的ORR。2022年ESMO大会公布的数据表明，在114例EGFR ex20ins且既往接受含铂化疗的NSCLC患者中（剂量递增和扩展队列28例，EXCLAIM延展队列86例），IRC评估的cORR为28%（95%CI: 20%-37%），研究者评估的cORR为35%（95%CI: 26%-45%）；IRC评估的DCR为78%（95%CI: 69%-85%），IRC评估中位缓解持续时间（duration of response, DOR）为15.8个月（95%CI: 7.4-19.4）；IRC评估的中位PFS为7.3个月（95%CI: 5.5-9.2），中位OS为20.2个月（95%CI: 14.9-25.3）。

2021年9月，FDA加速批准莫博赛替尼用于治疗铂类化疗期间或之后病情进展、经FDA批准检测方法证实为EGFR ex20ins突变的局部晚期或转移性NSCLC患者。同时，莫博赛替尼于2020年经国家药品监督管理局（National Medical Products Administration, NMPA）纳入“突破性治疗药物”和“优先审评”程序，目前已于2023年1月11日获NMPA批准用于临床治疗含铂化疗期间或之后进展且携带EGFR ex20ins的局部晚期或转移性NSCLC成人患者^[52]^。

莫博赛替尼用于一线EGFR ex20ins复发或转移NSCLC的研究正在探索中。EXCLAIM-2（NCT04129502）是一项随机化、国际多中心、开放性III期临床研究^[53]^（表4）。入选人群为一线EGFR ex20ins的复发或转移NSCLC。目标样本量为318例受试者。入组受试者将接受莫博赛替尼或含铂化疗。研究结果暂未公布。

##### 3.2.1.2 Amivantamab

Amivantamab是一种全人源的靶向EGFR和Met的双特异性抗体^[54]^。Amivantamab于2021年5月经FDA加速获批用于EGFR ex20ins且既往接受含铂化疗的NSCLC。该适应证获批基于一项I期临床研究（CHRYSALIS, NCT02609776）结果（表4）。该研究纳入了81例EGFR ex20ins且既往接受含铂化疗的NSCLC，受试者接受Amivantamab 1,050 mg（基线体重<80 kg）或1,400 mg（基线体重≥80 kg）静脉输注，前4周qw、4周后q2w治疗。主要有效性终点为IRC评估的ORR。2022年美国临床肿瘤学会（American Society of Clinical Oncology, ASCO）大会上公布的数据^[55]^表明，IRC评估的cORR为39.5%（95%CI: 28.8%-51.0%），研究者评估的cORR为35.8%（95%CI: 25.4%-47.2%），IRC评估的中位DOR为11.1个月（95%CI: 6.9-NE），IRC评估的中位PFS为8.3个月（95%CI: 6.5-10.9）；中位OS为22.8个月（95%CI: 14.6-NE）。

Amivantamab用于一线EGFR ex20ins复发或转移NSCLC的研究正在探索中。PAPILLON（NCT04538664）是一项随机化、开放性III期临床研究^[56]^（表4）。入选人群为EGFR ex20ins的一线晚期NSCLC。目标样本量为300例受试者。入组受试者将接受Amivantamab联合含铂化疗或仅用含铂化疗。研究结果暂未公布。

#### 3.2.2 I期/II期研究

##### 3.2.2.1 波齐替尼

波齐替尼是一种新型EGFR-TKIs，能够不可逆地阻断EGFR、HER2以及HER4的信号通路，从而使过表达上述受体的肿瘤细胞增殖得到抑制^[57]^。

ZENITH20研究（NCT03318939）是一项波齐替尼用于治疗EGFR ex20ins突变NSCLC的II期研究（表4）。该研究中，队列1纳入了EGFR ex20ins且既往接受含铂化疗的NSCLC，队列3纳入了一线EGFR ex20ins的晚期或转移NSCLC。以上受试者均接受波齐替尼16 mg qd。主要有效性终点为ORR。研究结果显示，队列1（既往治疗组）共入组115例受试者，ORR为14.8%（95%CI: 8.9%-22.6%），DCR为68.7%（95%CI: 59.4%-77.0%），中位DOR为7.4个月，中位PFS为4.2个月^[58]^。队列3（一线治疗组）共入组79例受试者，cORR为27.8%（95%CI: 18.4%-39.1%），DCR为86.1%，中位DOR为6.5个月，中位PFS为7.2个月^[59]^。值得注意的是，综合有效性和安全性数据，目前波齐替尼已经主动终止了在中国的临床注册研究，暂停了在中国的进一步研发。

##### 3.2.2.2 舒沃替尼（DZD9008）

舒沃替尼是一款新型选择性、不可逆EGFR抑制剂，通过抑制表皮生长因子作用进而诱导癌细胞凋亡。基于WU-KONG1（NCT03974022）和WU-KONG2（CTR20192097）研究，经NMPA、FDA“突破性疗法认定”^[60]^。

WU-KONG6研究（CTR20211009）是一项舒沃替尼治疗EGFR ex20ins突变NSCLC的II期研究（表4）。该研究受试者为EGFR ex20ins且既往接受含铂化疗的晚期NSCLC，接受舒沃替尼300 mg qd治疗。主要研究终点为盲态独立中心审查（Blinded Independent Central Review, BICR）评估的ORR。截至2022年7月31日，共纳入97例受试者。研究结果表明BICR评估的cORR为59.8%，脑转移患者的ORR为48.4%。所有患者的DOR尚未达到^[60]^。

##### 3.2.2.3 TAS6417（CLN-081）

CLN-081（TAS6417）是一种口服的不可逆EGFR抑制剂，具有独特的支架结构，其特异性支架可安装在EGFR铰链区ATP结合位点上，对EGFR突变（包括EGFR ex20ins）具有良好的广谱活性^[61]^。一项多中心I期/IIa期研究（NCT04036682）对CLN-081在EGFR ex20ins且既往接受治疗的晚期NSCLC中的疗效进行了评估（表4）。在剂量爬坡过程中探索了包括30 mg、45 mg、65 mg、100 mg和150 mg bid在内的剂量水平，并以30 mg、65 mg和100 mg bid开始疗效扩展。IIa期研究在基于方案规定的安全性和有效性标准之上，以100 mg bid剂量扩展，主要疗效终点为剂量扩增队列的ORR。2022年ASCO大会上公布的结果^[62]^表明，截至2022年5月9日，共73例受试者接受30 mg-150 mg不同剂量的CLN-081，确认部分缓解（confirmed partial response, cPR）率为38.4%，DOR为10个月，中位PFS为10个月（95%CI: 6-12）。

此外，多种EGFR ex20ins特异性靶向药物（AP-L1898、HS-10376、LNG-451等，表4）用于NSCLC治疗也已进入临床探索阶段，虽目前暂无相关临床数据。

### 3.3 其他治疗

针对EGFR ex20ins的其他新型治疗方式也在探索中。Luminespib（NVP-AUY922）是一种热休克蛋白90（heat shock protein 90, HSP90）抑制剂。临床研究^[63]^显示Luminespib在EGFR ex20ins且既往经治的晚期NSCLC中ORR为17%。Tarloxotinib是一种低氧激活的pan-HER激酶抑制剂，能够在病理生理缺氧情况下释放Tarloxotinib-E，后者对EGFR ex20ins NSCLC也有一定疗效获益潜力^[65]^。

## 4 展望

在EGFR突变患者中，EGFR ex20ins突变频率仅次于EGFR ex19del和L858R，且具有多种亚型，在庞大的肺癌患者基数下，每年新发患者数目不容小觑。但由于EGFR ex20ins突变特殊的分子结构限制了传统TKIs治疗的疗效，导致应答率较低，目前一线治疗方式仍以化疗为主。正在进行的临床试验^[66]^的结果表明，患者经过EGFR ex20ins突变检测后，应用新型TKIs药物进行靶向EGFR ex20ins的治疗效果能够达到更好的临床治疗效果。FDA已批准莫博赛替尼与Amivantamab用于含铂化疗失败或进展后的EGFR ex20ins突变NSCLC患者，莫博赛替尼也于近日获得NMPA正式批准。

针对EGFR ex20ins检测的重要性逐步提高。现阶段经NMPA批准的检测NSCLC相关基因突变的方法很多，各有其优势和局限性。实时荧光定量PCR为临床常用检测技术之一，灵敏度相对较高，操作简便，易于在临床开展。但针对EGFR ex20ins这类异质性较强的突变，PCR覆盖亚型有限，在临床应用过程中可能造成患者漏检，40%-50%患者无法检测到该突变^[38,39]^。NGS检测通量高，灵敏度高，可检测未知突变位点，并能覆盖EGFR ex20ins所有位点，理论上可检出所有EGFR ex20ins亚型。但NGS检测对样本要求高，操作步骤复杂，耗时长，数据分析要求高，成本相对也高。因此，临床广泛应用NGS仍具有挑战^[44]^。

随着EGFR ex20ins靶向药物可及性的不断提高，EGFR ex20ins检测的全面性和重视度有待进一步改善。国内外指南均建议对晚期含腺癌成分的NSCLC常规进行EGFR ex20ins基因检测^[5,31,35]^。在临床实践中，医师在提供患者治疗方案之前应让患者接受EGFR ex20ins基因突变检测，从而推动NSCLC精准靶向治疗的不断进步。

为进一步提高NSCLC精准诊断和个体化治疗水平，液体活检是组织活检的一个重要补充。未来利用血液循环肿瘤DNA（circulating tumor DNA, ctDNA）动态监测EGFR ex20ins突变患者靶向治疗过程中肿瘤分子标志物的变化，特别是用于探索新出现的耐药相关基因变异将是重要方向。

各实验室应该根据自身情况，选择相应的PCR或NGS检测平台并应考虑药物可及性，以更全面准确地反映患者基因突变的状态为目标，从而更好地给予患者个体化治疗方案选择^[25]^。
